# Dip-pen patterning of poly(9,9-dioctylfluorene) chain-conformation-based nano-photonic elements

**DOI:** 10.1038/ncomms6977

**Published:** 2015-01-19

**Authors:** Aleksandr Perevedentsev, Yannick Sonnefraud, Colin R. Belton, Sanjiv Sharma, Anthony E. G. Cass, Stefan A. Maier, Ji-Seon Kim, Paul N. Stavrinou, Donal D. C. Bradley

**Affiliations:** 1 Department of Physics and Centre for Plastic Electronics, Imperial College London, London SW7 2BZ, UK; 2 Department of Physics, Imperial College London, London SW7 2BZ, UK; 3 Department of Chemistry and Institute for Biomedical Engineering, Imperial College London, London SW7 2AZ, UK

## Abstract

Metamaterials are a promising new class of materials, in which sub-wavelength physical structures, rather than variations in chemical composition, can be used to modify the nature of their interaction with electromagnetic radiation. Here we show that a metamaterials approach, using a discrete physical geometry (conformation) of the segments of a polymer chain as the vector for a substantial refractive index change, can be used to enable visible wavelength, conjugated polymer photonic elements. In particular, we demonstrate that a novel form of dip-pen nanolithography provides an effective means to pattern the so-called β-phase conformation in poly(9,9-dioctylfluorene) thin films. This can be done on length scales ≤500 nm, as required to fabricate a variety of such elements, two of which are theoretically modelled using complex photonic dispersion calculations.

## Abstract (web summary)

The optoelectronic properties of semiconducting polymers are controlled by altering chemical structure and/or inter-chain order. Perevedentsev *et al*. propose a nanopatterning approach whereby the geometry of polymer chain segments is modified to engineer metamaterial structures for visible light.

## Graphical abstract

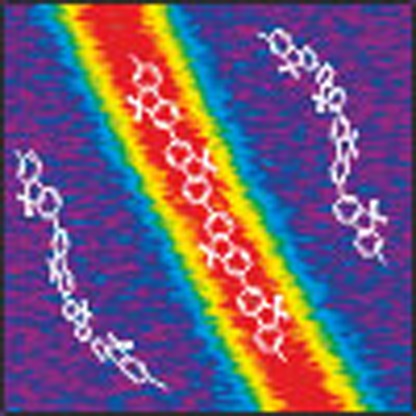

The bottom-up, molecular approach to structure formation offers interesting opportunities for metamaterials fabrication without the need for conventional optical or electron beam lithography, an important consideration where visible wavelength metamaterials, requiring nano-structuring, are concerned. This should not be confused with the molecular analogue approach to structured-metal-based, metamaterial design and function^1^. Typical embodiments use molecular assembly to generate a template to guide metal particle array formation or the attachment of molecular units to metal nanoparticles that enables their assembly into hierarchical structures. In the former category, interesting recent work includes metal-organic framework structures^2^ and in the latter, bio-enabled assembly strategies^3^. However, these approaches do not use the optical properties of the molecular component to define the interaction with electromagnetic radiation; that interaction is still based on metals. In parallel, graphene-based molecular structures and carbon nanotubes have been considered theoretically as replacements for the split ring and other basic units of traditional metal metamaterials design but their assembly into suitable arrays was not discussed^4^.

We have previously introduced the concept of conformational metamaterials in which the physical geometry (conformation) of a molecule acts as a vector for refractive index change. Our initial proof-of-concept publication^5^ demonstrated that masked solvent vapour exposure could then be used to define millimetre-scale spatial patterns of varying metamaterial content and hence refractive index (and photoluminescence (PL) emission colour) within a planar film. The solvent-diffusion-controlled pattern resolution led to a pseudo-Gaussian boundary index profile and performing the patterning on top of a one-dimensional grating structure allowed spatial selection of distributed feedback (DFB) lasing wavelength^5^, in essence a form of frequency-selective surface. In a subsequent publication, the patterning resolution was improved to 250 μm using a scanned, nozzle-based solvent vapour printing method^6^.

We now demonstrate the formation of spatial patterns of the β-phase conformation in poly(9,9-dioctylfluorene) (PFO) on length scales ≤500 nm using a novel dip-pen nanolithography (DPN) method, in which a liquid solvent is used as ink. Unlike conventional DPN techniques in which patterning involves deposition of material onto or its removal from a substrate, here the solvent ink temporarily resides in contact with/swells an existing film and thereby induces the required local polymer chain segment conformation change. Further reductions in length scale are anticipated, offering the prospect of a versatile approach to visible wavelength, conformation-based, conjugated polymer photonic elements.

## Results

### DPN ink selection for β-phase generation

**Figure 1:**
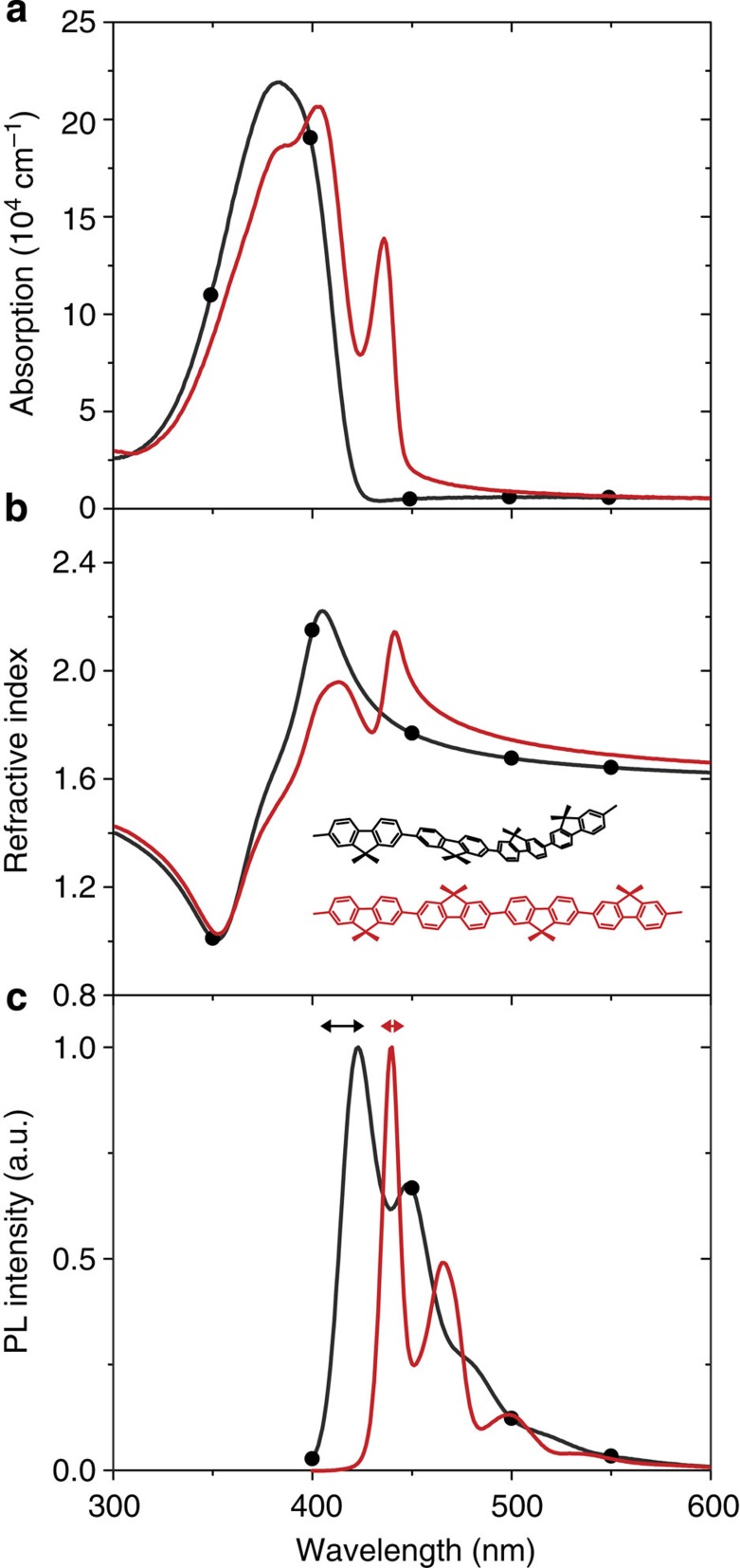
Optical properties of PFO thin films. (**a**) Absorption, (**b**) calculated refractive index and (**c**) peak-normalized PL spectra of a glassy PFO film (black lines+circles) spin-coated from hot toluene onto a heated fused silica substrate and the same film after it has been structured by immersion in decalin and dried under ambient conditions over several hours (red lines). The horizontal arrows in **c** indicate the spectral position and band-pass of the confocal microscopy filters used to preferentially select glassy (415 nm) and β-phase (440 nm) PL (*vide infra*). The inset in **b** shows schematic representations of glassy (black) and β-phase (red) chain segments with their alkyl side-chains (C_8_H_17_) omitted for clarity.

Spin-coating PFO thin films from good solvents (Hildebrand parameter *δ*≈9.1–9.3 cal^1/2^ cm^−3/2^)^7^,^8^ with low-boiling points, such as tetrahydrofuran (*δ*≈9.1, BP=66 °C)^9^ and chloroform (*δ*≈9.3, BP=61 °C)^9^, typically results in glassy films that are isotropic within the plane^10^. PFO chains in these films possess a broad distribution of torsionally connected molecular conformations that results in inhomogeneously broadened absorption with correspondingly un-resolved vibronic structure and, to a lesser degree (because of excitation migration before emission), PL^11^,^12^,^13^. Similar results can be obtained using toluene (*δ*≈8.9, BP=110 °C)^9^, especially when, as here, solution and substrate are heated before coating (Fig. 1). These glassy films form the baseline samples to which metamaterial physical structuring is then applied but unlike traditional metal-based systems this structuring does not rely on removal of the baseline material.

The distinct β-phase molecular conformation, which we use as our metamaterial vector for refractive index change, can be induced in glassy films by a variety of methods that apply stress to the polymer backbone, including differential thermal expansion^7^,^8^,^12^ and solvent vapour treatment^5^,^6^,^11^,^13^. The β-phase conformation comprises a rigid, chain-extended structure with an inter-monomer torsion angle of ~180°, such that the octyl substituents of neighbouring fluorene units lie on alternating sides of the polymer backbone^7^,^8^,^11^,^12^,^13^. Winokur and colleagues have predicted a specific alignment of these alkyl chains, alongside the conjugated backbone^14^. Presence of the β-phase conformation is readily identified by the resulting changes in optical properties. Its characteristic spectral features comprise a new red-shifted, clearly resolved absorption peak, enhanced vibronic structure and a small Stokes shift between absorption and PL emission (Fig. 1).

In addition to thermal stress and solvent vapour treatment, it has been demonstrated that swelling induced by dipping glassy PFO films into solvent–non-solvent mixtures, such as THF–methanol^15^,^16^ or toluene–methanol^16^, for tens of seconds at a time can generate β-phase chains with no appreciable dissolution of the film. These observations motivated our patterning strategy to use a DPN instrument to deliver small quantities of solvent ink to the surface of a glassy PFO film in order to locally induce the β-phase conformation to thereby define a desired photonic element. Key parameters to control in order to tune the magnitude of the index change and its lateral resolution are the swelling stress, the solvent residence time and the tendency of the solvent to spread on the film surface. However, given that most solvent mixtures are not azeotropic, it was decided to initially use a single solvent rather than a mixture to avoid the complications associated with changes in the mixture ratio during drying. After considering a number of possibilities, we selected decalin (decahydronaphthalene) as our DPN solvent ink. Decalin (*δ*=8.7 cal^1/2^ cm^−3/2^; BP≈190 °C)^9^ should be a moderately good solvent for PFO and its high boiling point will aid long retention times under ambient conditions.

In order to confirm the solvent’s suitability for generating β-phase chain segments, optical absorption and PL spectra were recorded for glassy PFO films before and after uniform immersion in decalin. The results are shown in Fig. 1a,c for a film of ~90 nm thickness. The glassy film shows a typical inhomogeneously broadened absorption band and a PL spectrum with partially resolved vibronic peaks^7^,^8^,^13^. After decalin immersion, the film shows strong β-phase absorption features at 436 and 403 nm and the characteristic red-shifted β-phase PL spectrum with well-resolved vibronic peaks at 440, 466, 499 and 532 nm (refs 7, 8, 13). Immersion in decalin clearly yields a high fraction of β-phase chain segments, with the 436 nm absorption peak as-strong if not stronger than previously reported^11^,^13^,^17^, confirming that decalin should be a good choice as DPN solvent ink.

The refractive index spectra, obtained from the corresponding absorption spectra using a Kramers–Kronig analysis^18^, are shown in Fig. 1b. The introduction of β-phase chain segments following decalin soaking results in a distinct increase in refractive index relative to the baseline glassy film: 10.9% at 450 nm falling to 5.5% at 475 nm, 4.1% at 500 nm and 2.2% at 600 nm. The utility of such index changes has previously been demonstrated for large-scale (relative to emission wavelength) photonic environments^5^,^6^. However, as modelled below, patterning of the β-phase regions on a *sub-micron* scale enables considerable innovation in the photonic element architecture.

### β-Phase patterning via DPN

DPN^19^,^20^ is a versatile technique that allows the precise deposition (feature sizes down to 100 nm) of a range of materials from ink-immersed atomic force microscopy (AFM) tips onto a variety of substrates using several different strategies. For example, direct writing of conducting polymer solution inks produces conductive feature sizes down to ~600 nm on substrates including polyethylene terephthalate and polydimethylsiloxane^21^, and composite inks comprising a pre-polymer carrier fluid and a starburst oligofluorene truxene molecule can be cured after deposition to generate arrays of fluorescent microdomes on silica on silicon substrates^22^. An alternative approach is to use DPN inks to etch patterns into a polymer resist that then acts as a template for metal nanostructure fabrication^23^.

**Figure 2:**
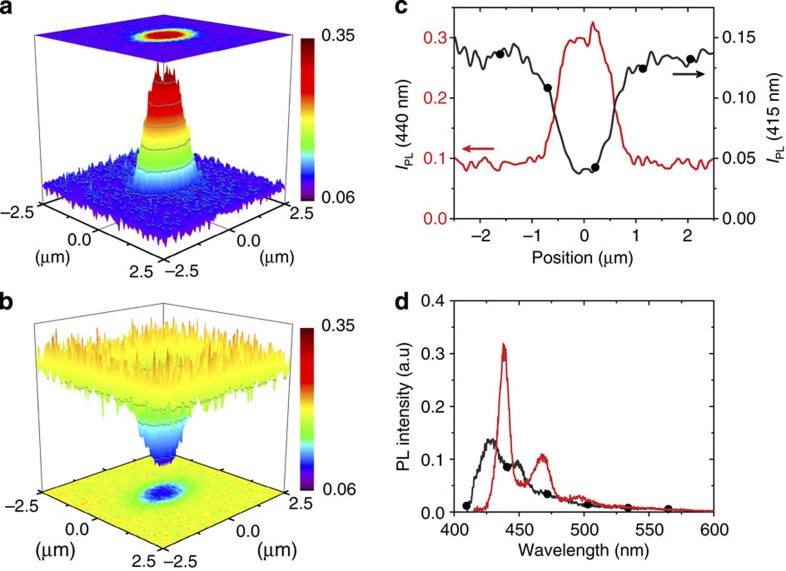
DPN patterning and imaging of a β-phase dot. Confocal PL images of a β-phase dot element, patterned by DPN using decalin ink and a dwell time of 20 s, recorded with PL filtered at (**a**) 440 nm and (**b**) 415 nm. PL intensity profiles, *I*_PL_(λ), obtained by taking a slice through the centre of the dot in the digitized image and presented here before deconvolution, are given in (**c**) with filtering at 440 nm (red line) and 415 nm (black line+circles). PL spectra acquired from the dot (red line) and the surrounding film (black line+circles) are shown in (**d**), with the respective intensities at 440 nm set to the average PL intensities measured on/off the β-phase dot in **a**.

Our approach to the formation of β-phase patterns on sub-micron length scales is based on a novel DPN strategy, in which a liquid solvent is used as ink. Unlike the conventional DPN techniques^21^,^22^,^23^, here a small amount of the solvent ink only temporarily resides in contact with the polymer film. The accompanying swelling of the film assists a fraction of chain segments to adopt the β-phase conformation. The metamaterial structuring is thereby expected to occur simultaneously with photonic element patterning. Test elements comprising arrays of dots were written with decalin ink as described in the Methods section. Confocal PL microscopy was used to probe their properties by filtered collection of emission light (cf. horizontal arrows in Fig. 1c) selected to be predominantly characteristic of the glassy polymer film (415 nm) or β-phase chain segments (440 nm); Fig. 2 presents typical results.

The images shown in Fig. 2a,b clearly demonstrate the local formation of β-phase chain segments as a result of DPN-deposition of decalin droplets onto the surface of a glassy PFO film. Photonic element patterning is thus indeed achieved simultaneously with metamaterial structuring. Filtering the PL at 440 nm (β-phase 0-0 vibronic, Fig. 2a) reveals a strong enhancement in detected intensity within the dot location. Conversely, filtering at 415 nm (glassy film 0-0 vibronic, Fig. 2b) shows a strong quenching in detected PL intensity within the dot. Corresponding 440 nm (red line) and 415 nm (black line+circles) PL intensity line profiles are shown in Fig. 2c. Both intensity profiles are smooth and continuous within the spatial resolution of the imaging system and reveal no abrupt drops in intensity at the centre of the dot. On the basis of this, we infer that the DPN dot-patterning process leads to negligible film scratching. Spectral data (Fig. 2d) acquired at the dot location (red line) and from the surrounding area (black line+circles) are also wholly consistent. The PL emission from the dot is entirely β-phase in origin while the surrounding film shows a typical glassy spectrum, with partial self-absorption of its 0-0 vibronic (cf. Fig. 1a,c).

The dimensions of the patterned β-phase dot element can be estimated from the 440 nm line profile in Fig. 2c following deconvolution of the imaging system’s point spread function (PSF). The full-width at half-maximum (FWHM) dot diameter *d* is deduced here to be ~1.05 μm.

### DPN process parameters

Adjusting the DPN process parameters allows control over the size of the patterned β-phase elements. The dwell time for contact between the DPN tip and the polymer film surface strongly affects the dot diameter that can be written; larger dots are formed for longer dwell times. Reducing dwell time to 1 s produced β-phase dots with a minimum diameter *d*≈600 nm, as deduced from the FWHM of 440 nm filtered PL profiles following PSF deconvolution. The observed dependence is common for DPN and results from the ink transferred to the film being essentially proportional to dwell time^20^,^24^,^25^.

Another factor that we have found to affect the size of the β-phase dot elements is the tip-to-film separation distance (that is, contact strength) maintained during patterning. The size and contrast of features decreased with increasing tip-to-film separation distance because of an associated hindrance of ink transport from the tip to the film surface; eventually no patterns could be discerned. Our observations are again consistent with numerous earlier experimental and theoretical studies of DPN patterning^20^,^25^,^26^,^27^. Given, however, the nature of the patterning process we have adopted, in addition to element size, the relative fraction of β-phase chain segments generated and hence the metamaterial refractive index increment can also be tuned, although not wholly independently of size. The efficient Förster resonant excitation transfer from glassy to β-phase chain segments conversely ensures that the PL emission spectrum switches abruptly once a percent or few of β-phase segments are formed^7^,^11^,^12^,^13^,^14^,^15^.

**Figure 3:**
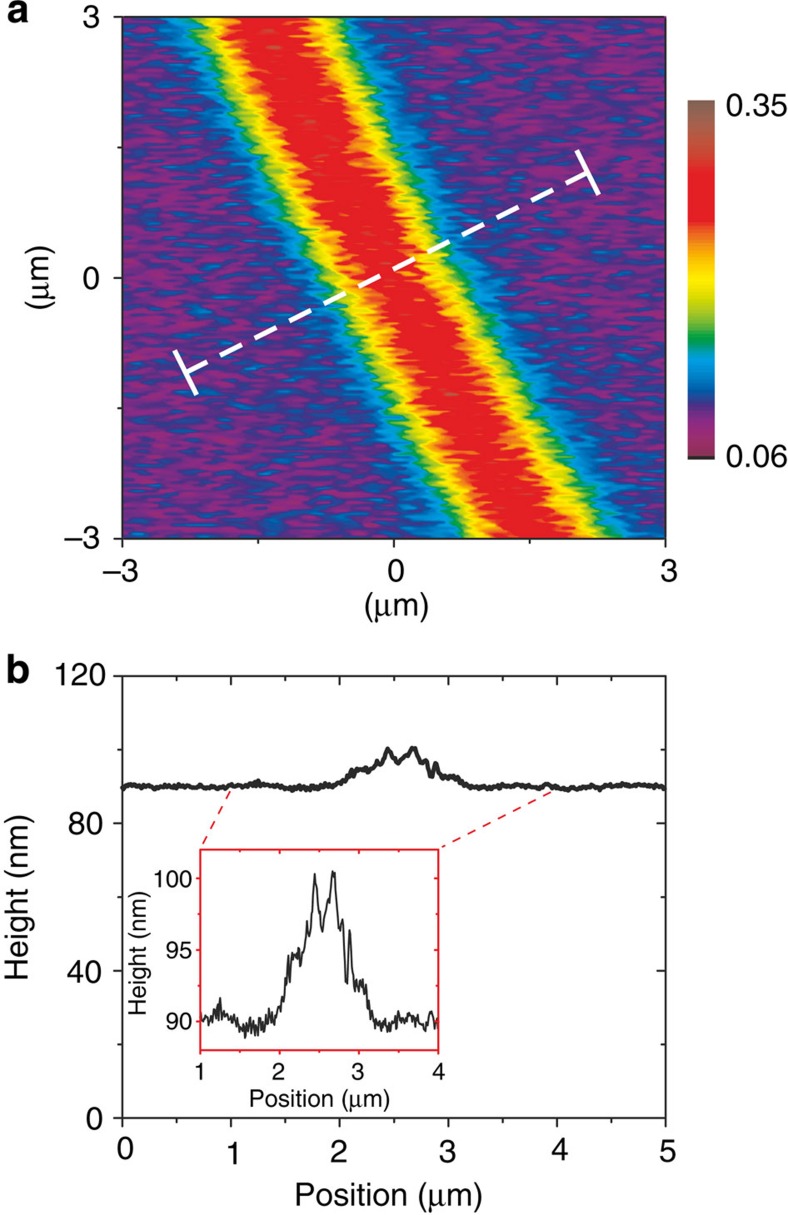
DPN patterning and imaging of continuous β-phase stripes. (**a**) Confocal PL image of a β-phase stripe element, DPN patterned using decalin ink at a writing speed of 0.1 μm s^−1^. The image was recorded with PL filtered at 440 nm. (**b**) AFM topographic profile measured along the path indicated by the dashed line in **a**. The main panel shows the full film thickness profile, whereas the inset provides a magnified view.

As discussed in more detail below, β-phase stripe elements are also of interest for applications such as waveguides (light propagation along a stripe) or refractive index gratings (light propagation normal to a set of stripes). We have consequently investigated methods to write such stripes. Figure 3a shows a confocal PL image (recorded with 440 nm filtered detection) for a stripe written continuously with decalin ink at 0.1 μm s^−1^ DPN tip-speed across the surface. The associated PL intensity cross-section (not shown) yields, after PSF deconvolution, a FWHM width, *w*≈1 μm.

The corresponding effect of DPN patterning on film topography was investigated by AFM; Fig. 3b shows a typical film thickness profile across the β-phase stripe element presented in Fig. 3a. The average height of the patterned region increases by ~5 nm relative to the surrounding film, likely as a result of chain reorganization. The root-mean-square roughness values for the pristine and patterned regions are 0.5 and 3.1 nm, respectively. An increase in film roughness is reportedly linked closely with the formation of high β-phase fractions because of increasing chain aggregation^17^, an effect that may be partly responsible for the observations in Fig. 3b; tip–film interactions will also be important.

We note, however, that the β-phase patterning-induced changes in topography are rather small; the change in height corresponds to only 6% of film thickness, whereas the increase in film roughness is equally minor. Moreover, were it to prove necessary to reduce any associated scattering, remedial action in the form of mechanical rolling or lamination of a thin overlay could be implemented. Further optimization of the DPN method should also be explored. Additional information on film topography is presented in Supplementary Fig. 1.

**Figure 4:**
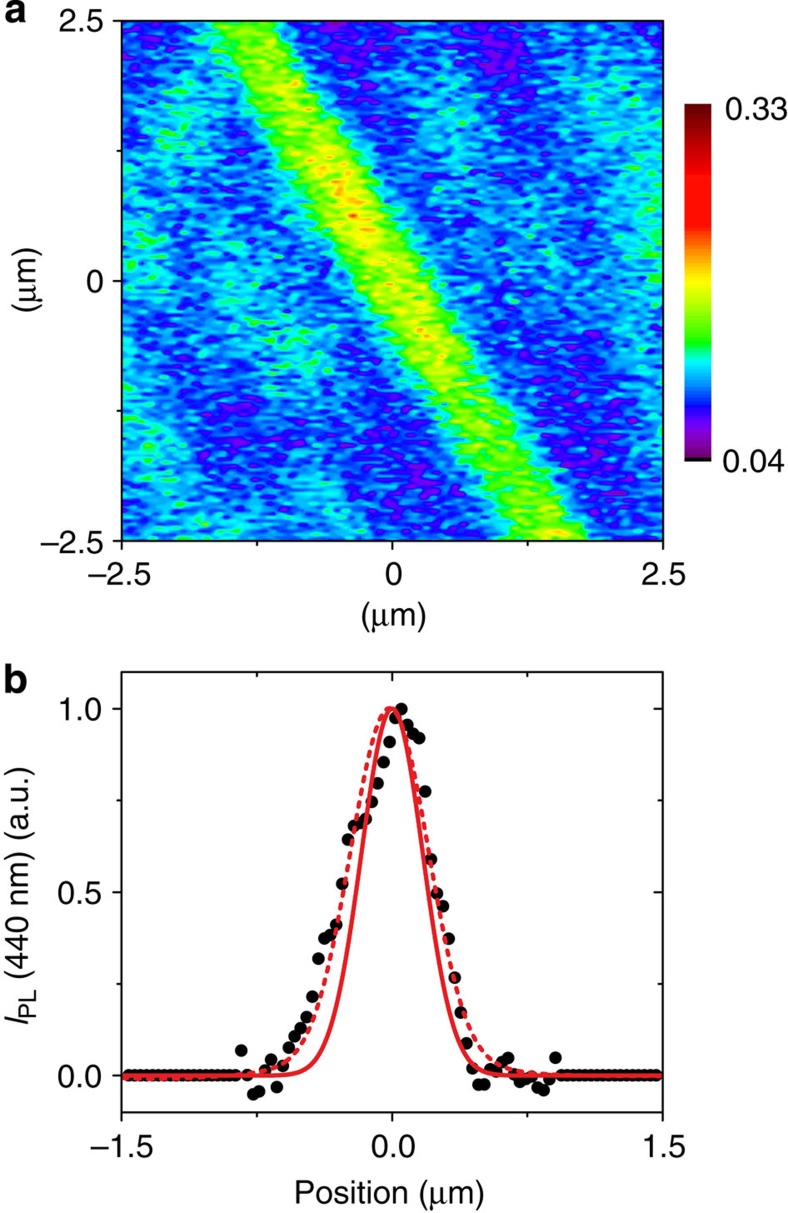
DPN patterning of β-phase stripes using low-boiling point solvents. (**a**) Confocal PL image of a β-phase stripe element, DPN patterned at 0.1 μm s^−1^ writing speed using a low-boiling point cyclohexane/IPA 3:1 (vol/vol) mixture ink. The image was recorded with PL filtered at 440 nm. (**b**) Peak-normalized PL intensity cross-section, *I*_PL_ (black circles), obtained by taking a slice through the centre of the line in the digitized image. The convolution of a Gaussian ‘true’ β-phase profile (solid red line; FWHM=400 nm) with the PSF (not shown; FWHM=340 nm) produces an ‘observed’ PL intensity profile (dashed red line) that fits the experimental data.

The dimensions of the β-phase photonic elements are understood to depend strongly on the lateral diffusion/dispersion of the solvent ink, facilitated by the high boiling point of decalin. Consequently, we tested a second ink, comprising a lower boiling point, 3:1 (vol/vol) mixture of cyclohexane (*δ*≈8.2 cal^1/2^ cm^−3/2^, BP≈81 °C)^9^ and isopropyl alcohol (IPA; *δ*≈11.5 cal^1/2^ cm^−3/2^, BP≈83 °C)^9^ that has previously also been successfully used to structure β-phase chain segments in glassy PFO films by immersion^15^. The corresponding PL image for a stripe element patterned with this ink at the same 0.1 μm s^−1^ DPN tip-speed is shown in Fig. 4a. The PSF-deconvolved PL intensity cross-section of the β-phase stripe (cf. Fig. 4b) shows the anticipated reduction in FWHM width to *w*≈400 nm. A detailed description of PSF deconvolution and estimation of conformational pattern dimensions is given in Supplementary Note 1. Interestingly, a lower contrast in detected PL intensity is observed for the stripe in Fig. 4a relative to the decalin-patterned stripe image in Fig. 3a, indicating a reduced β-phase fraction from the more rapid evaporation of solvent and heralding a potential trade-off between feature size and index contrast. More work will be needed to assess this situation.

### DPN-patterned β-phase photonic element architectures

With the sub-micrometre patterning of metamaterial β-phase PFO elements firmly demonstrated, there emerge a number of photonic structures that can exploit the attendant refractive index changes at this length scale. Perhaps the simplest, and one that extends from previous proof-of-concept work^5^,^6^, is that β-phase stripes formed within a glassy baseline film provide the basis for simple waveguide structures that, with lateral line widths now <1 μm, can support single *transverse* modes. In addition, structures that offer a more sophisticated photonic environment are possible and we numerically examine two variants that build on the results presented in Figs 2, 3, 4, details of this modelling are presented in Supplementary Fig. 2 and Supplementary Note 2.

The first is a one-dimensional photonic crystal lattice comprising an array of parallel DPN-patterned β-phase stripes arranged periodically with spacing *Λ* matched to the PFO PL spectrum. The resulting diffraction grating is one in which physical corrugations of the film are minimal and therefore refractive index modulation (phase) is key. From the point of view of modal propagation within the film, the structure is in a class of open periodic optical waveguides; these have considerable interest for applications requiring distributed Bragg reflectors, DFB lasers, beam steering devices as well as output and input couplers^28^.

**Figure 5:**
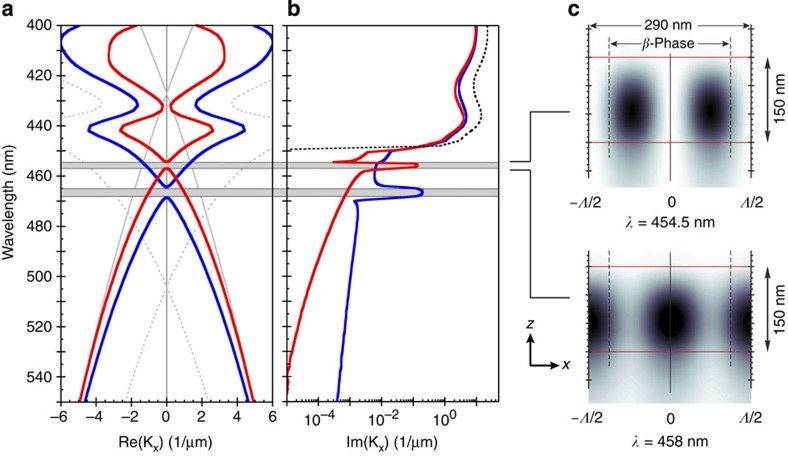
A thin-film chain-conformation-based photonic grating structure. Full, complex dispersion calculations for the TE- and TM-guided modes are shown for a 150-nm-thick film, periodically patterned with alternating stripes of β- and glassy-phase PFO. A period of *Λ*=290 nm was selected with the β- and glassy-phase regions spanning, respectively, 75% and 25%. In (**a**), the modal dispersion, displayed in a reduced zone scheme, highlights the distinct band gaps (horizontal grey bars) for both TE (blue) and TM (red) modes. Propagation losses for both modes are shown in (**b**) along with the absorption in the β-phase region (dotted line). The unit cell of the photonic structure is shown in (**c**), with the upper and lower red horizontal lines indicating film interfaces with air and the substrate, respectively. The vertical solid black line delineates the centre of a single period and the vertical dashed lines delineate the edges of the β-phase stripe. The stripe long axis runs into the page, in the *y*-direction. The illustrative calculated field distributions (|**H**_y_(x,z)|^2^) at 454.5 and 458 nm (darker=larger modulus) clearly reveal the characteristic standing wave patterns (and their spatial displacement) for wavelengths selected to lie on either side of the TM band gap.

A pertinent question is to what degree the refractive index contrast between β-phase structured and baseline glassy PFO is of practical interest in this context. Our analysis, based on modelling, suggests that it can readily satisfy typical requirements. As an example, the full (complex) photonic dispersion for transverse electric (TE) and transverse magnetic (TM) modes propagating in a 150-nm thickness PFO film with periodically patterned β-phase stripes (*Λ*=290 nm, 75% fill factor, β-phase fraction as for the decalin-immersed reference film shown in Fig. 1) is displayed in Fig. 5.

In this reduced zone picture, clear band gaps appear at **k**_x_=0 for both TE and TM modes as a result of successful contra-coupling between the Floquet-Bloch space harmonics of the guided modes (Fig. 5a,b). These specific band gaps (at **k**_x_=0) are routinely used to instigate surface emitting laser action for organic gain media within so-called second-order DFB geometries where all but one of the space harmonics couple together to provide resonator action^29^,^30^. The calculated propagation losses, shown in Fig. 5b for both TE (blue line) and TM (red line) modes, reveal, as expected, spectrally localized enhancements coincident with the band gaps in Fig. 5a. This peaked modal attenuation, at wavelengths beyond the absorption of the β-phase material (dotted line in Fig. 5b), defines the region of significant optical feedback. The resulting laser properties, such as threshold and slope efficiency, can be further tuned through film thickness, grating period and fill factor.

Additional evidence for the resonator properties of the modelled β-phase stripe grating structure is found in the field distributions calculated at band edge wavelengths as shown in Fig. 5c for the TM gap. At the band edges, the group velocity approaches zero, generally indicating an absence of real power transfer in the propagation direction. For second-order DFB geometries, this is only partly correct, with one harmonic not involved in the contra-coupling but rather in the radiative extraction of power out of the plane (cf. Supplementary Fig. 2b). Nevertheless there is sufficient coupling and pairing of the remaining space harmonics close to the band edges to form standing waves along the propagation direction as indicated in Fig. 5c for *λ*=454.5 and 458 nm. The clear spatial shift in the field distributions either side of the band gap is another characteristic feature of photonic structures^31^.

**Figure 6:**
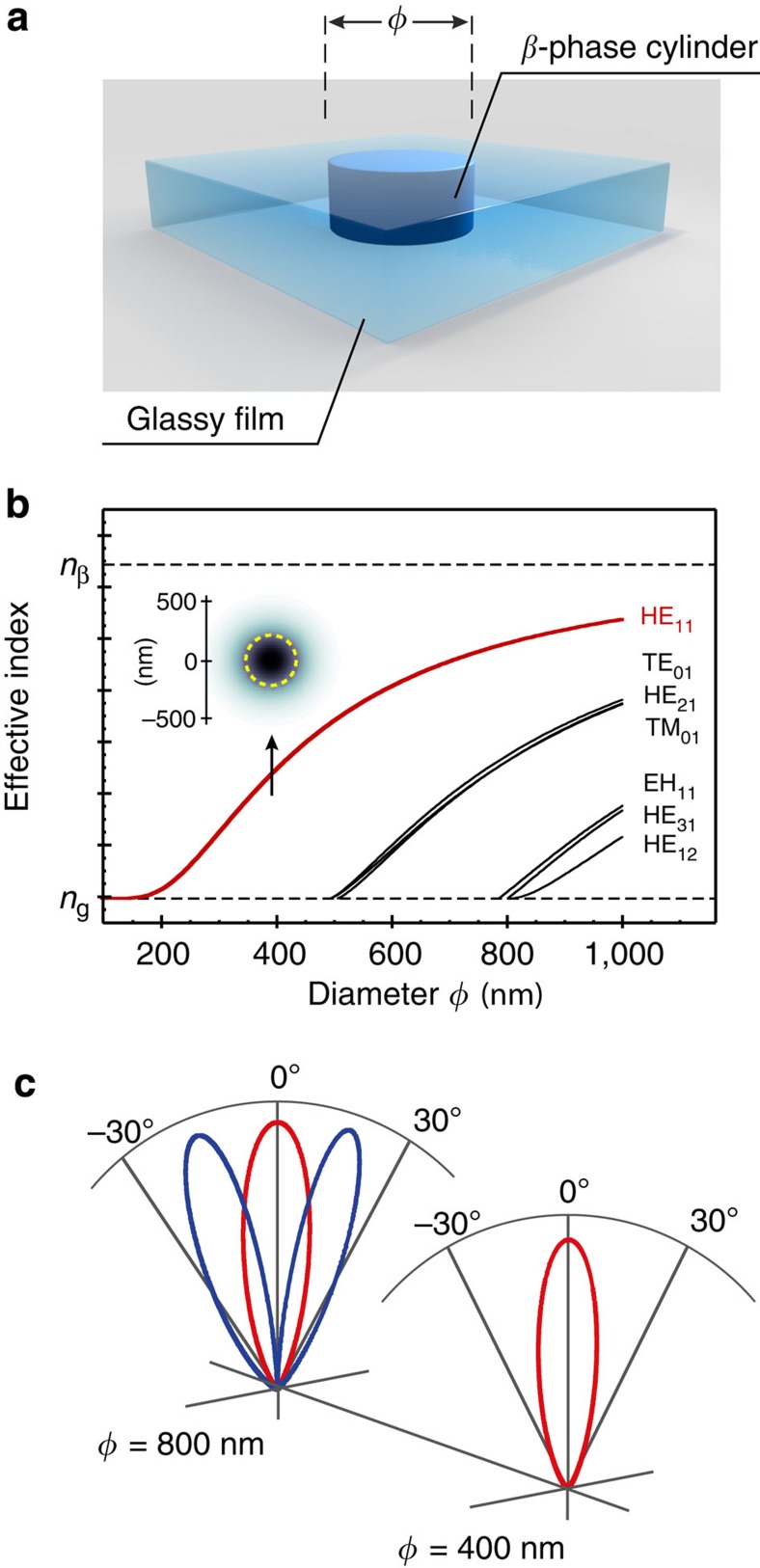
Propagation and radiation from β-phase photonic cylinders. (**a**) Illustration of a β-phase vertical cylinder of diameter *φ* embedded within a thin film of glassy-phase PFO. (**b**) Full-field dispersion of the supported optical modes propagating along the cylinder axis. The inset shows the intensity distribution (darker=higher) of the HE_11_ mode for *φ*=400 nm; the dashed circle delineates the cylinder cross-section and confirms the high degree of confinement. (**c**) Calculated far-field radiation profiles of HE_11_ (red) and HE_21_ (blue) guided modes emanating from the cylinder cross-sections at the thin film surface. Two cylinder diameters (*φ*=400 and 800 nm) are shown.

As a second example, we model the properties of two-dimensionally confined photonic structures based on the β-phase dot elements shown in Fig. 2. Assuming that the conformational changes propagate vertically through the whole thickness of the film, these are more accurately β-phase *cylinder* elements as shown schematically in Fig. 6a. In practice, one cannot easily be sure that the element has a uniform cross-section through the film but in our previous work^5^, spectroscopically tracking the Fabry–Perot resonances from films up to 400 nm in thickness, we found little evidence of inhomogeneities in the vertical distribution of β-phase chain segments; at least from an optical (refractive index) perspective. We have therefore approximated the structure as a well-defined cylinder of arbitrary length, terminated at the film–air and film–substrate interfaces.

For optical wavelengths coincident with the PL emission (that is, *λ*>440 nm), the system is reminiscent of a basic step-index optical fibre (albeit in reality a rather short one), comprising a β-phase structured core and a (semi-infinite) glassy cladding (with properties as per Fig. 1). Choosing a PL wavelength (460 nm) close to the β-phase S_1-0_ 0-1 vibronic peak yields *n*_core_=1.869 and *n*_cladding_=1.739. The resulting index step, Δ*n*≈7%, will clearly reduce for lower β-phase fractions but serves as a useful reference point for exploring application potential. Figure 6b shows the familiar modal dispersion for such a step-index fibre with light propagation normal to the film; data are shown as a function of cylinder diameter, *φ*. Only a single, highly confined, HE_11_ mode (cf. inset to Fig. 6b) is supported for *φ*<500 nm, whereas for larger *φ* more modes arise. For example, with *φ*=800 nm, HE_11_, TE_01_, HE_21_ and TM_01_ modes are all supported (cf. Fig. 6b). PFO chain segments with PL transition dipoles within the film plane can couple to these modes, leading to radiation profiles that are mode-specific and that consequently vary with *φ*. At the film top surface and for *φ*=400 nm, only the fundamental HE_11_ mode is supported and the energy emerges from the cylinder cross-section with an angular FWHM<30° (cf. Fig. 6c). This is considerably more directional than expected for the usual Lambertian emission from a plain surface (that is, typically with FWHM ~120°)^32^. Increasing the diameter of the β-phase cylinder increases the number of modes supported, each mode with its own far-field distribution, such that the overall angular width of the radiation profile increases. For example, for *φ*=800 nm, considering only the HE_11_ and HE_21_ modes leads to an angular FWHM ~60° (Fig. 6c). Although we have focused here on the effect of varying the cylinder diameter for a single emission wavelength, it is useful to recall that owing to the different dispersions of the discrete set of optical modes, chosen wisely a cylinder’s dimensions can also serve to angularly separate the spectral components of a typically broad conjugated polymer PL spectrum^33^.

This last example highlights the ability to use the refractive index contrast between β-phase structured and baseline glassy PFO to define effective apertures that control both the spatial and spectral properties of the emission from a uniform-chemical-composition planar thin film. In the case that the Δ*n* features have dimensions <1 μm, the resulting radiation profiles can become highly directional. One interesting implementation would be to write an array of such emitting apertures into the active layer of a polymer light emitting diode structure (see Supplementary Fig. 3 for the confocal PL image of a sequence of DPN-patterned dots).

## Discussion

There are two important questions that arise in considering the future development of our novel DPN approach to simultaneously structuring and patterning visible wavelength conjugated polymer-based conformational metamaterials.

First, does this method have a plausible generality beyond the specific example illustrated here of the generation of β-phase chain segments in PFO? The answer to this question is yes, noting in particular that similarly large changes in optical spectra can be induced by solvent vapour treatment of thin film samples of certain alkoxy-/alkyl-phenyl substituted polythiophenes such as poly(3-(2′-methoxy-5′-octylphenyl)thiophene)^34^. Further work will need to be done to design other suitably substituted conjugated polymer structures for which significant conformation-induced switching of optical response can be achieved but the prospects for this look very good.

Second, does this approach compare favourably with alternative nanopatterning methods? We note here that β-phase nano-patterns have also been reported using a copolymer comprising 9,9-dioctylfluorene and 9,9-divinyloxyethoxyhexylfluorene units^35^. The β-phase was generated uniformly in glassy films of this copolymer by exposure to toluene vapour and selective cross-linking of vinyl-ether groups was subsequently performed by scanned electron beam exposure. The films were finally developed with chloroform to leave a positive replica of the written pattern within which the β-phase conformation was retained. Clearly, our DPN process involves fewer steps and, advantageously, retains a planar film format. To return to such a format using the electron beam approach requires an additional back-filling step, as indeed performed by Kuehne *et al*. using the same copolymer spin-coated from a good solvent to yield the complementary baseline glassy phase. Again, therefore, the answer is yes and as a consequence we anticipate that DPN using solvent inks to simultaneously structure and locally pattern the conformation of conjugated polymer chain segments within thin film samples looks like an attractive approach to generating nanoscale photonic elements. Looking forward, it will be beneficial to be able to further reduce the feature sizes that can be routinely patterned; careful optimization of the DPN patterning process and surface chemistry are expected to be useful in that regard. In addition, the uniformity and scalability of the process remains to be established. We note, however, that, in general, DPN has proven to be a relatively scalable process, with patterning heads comprising arrays of thousands and even tens of thousands of tips sold commercially.

In summary, we demonstrate a novel approach to conformational-metamaterial-based photonic elements defined by simultaneous structuring and spatial patterning of the chain conformation in thin film PFO samples using local DPN delivery of selected solvent inks. Confocal microscopy of the resulting patterns (both arrays of dots and stripes with minimum obtained feature sizes of approximately 500 nm) reveals the expected changes in PL that accompany a switch from glassy- to β-phase chain segments, whereas AFM measurements show limited changes to film planarity, a desirable feature for ease of integration. Decalin was shown to be suitable as solvent ink for DPN patterning, capable of yielding a high β-phase chain segment fraction, and a number of processing parameters were optimized in order to reduce the dimensions of the patterned β-phase elements into the sub-micrometre scale. A lower-boiling-point cyclohexane/IPA (3:1 (vol/vol)) solvent-mixture ink was also used, allowing stripe line widths *w*≈400 nm to be written.

Our DPN approach is expected to provide a practical means to fabricate a diverse range of photonic element architectures. Detailed optical modelling was used to explore two specific examples, namely a one-dimensional grating comprising alternating structured β-phase and baseline glassy stripes and a cylindrical β-phase inclusion within an otherwise glassy film, and a number of attractive features were highlighted. Future work will involve DPN fabrication and optical characterization of a number of PFO-based devices and will explore alternative conformational-metamaterial classes including additional conjugated polymer systems.

## Methods

### Materials

PFO, synthesized by Suzuki coupling, was supplied by Cambridge Display Technology and used as received. The sample used for this study had a polystyrene-equivalent gel permeation chromatography measured number average molecular weight *M*_n_=28 × 10^3^ g mol^−1^, with polydispersity index=2.93. Decalin (decahydronaphthalene; anhydrous, ≥99%, mixture of *cis* and *trans* isomers) and IPA (anhydrous, 99.5%) were purchased from Sigma-Aldrich; HPLC-grade cyclohexane (≥99.9%) and toluene (≥99.7%) were purchased from VWR. All solvents were used as received.

### Thin film fabrication

Toluene solutions of PFO (15 g l^−1^) were spin-coated on fused silica substrates at 2,000 r.p.m. to yield 90- to 100-nm-thickness films. In order to ensure that these were high-quality glassy films, substantially free from β-phase chain segments (as evidenced by PL spectra), both the solution and substrates were placed on a hotplate at 100 °C for 2 min immediately before spin-coating. In order to test the potential of decalin as ink for DPN generation of β-phase chain segments, we flooded the top surface of glassy PFO films (previously spin-coated from hot toluene onto heated substrates) with the solvent and allowed it to slowly evaporate in a fume hood before characterizing the resulting optical properties.

### DPN structuring and patterning

DPN structuring and patterning was performed on a NanoInk NLP 2000 system, using Bio M pens. The combination of a relatively low pen spring constant (0.6 N m^−1^) and minimal tip-to-film contact ensured that little scratching of the polymer film occurred during writing. Decalin and cyclohexane/IPA (3:1 by volume) were loaded, in turn, into the inkwell reservoirs and the pens were inked for 5 s before writing. Typical dwell times for writing individual dots were 1–20 s and 4 × 4 arrays of dots were written to confirm reproducibility. Stripes were typically 40 μm in length and written at 0.1 μm s^−1^ DPN tip-speed. All DPN patterning was carried out under ambient conditions.

### Characterization

Absorption spectra were obtained for thin film samples on fused silica substrates using a dual beam Shimadzu UV-2550 spectrophotometer. The corresponding PL spectra were recorded in reflection geometry using a Horiba Fluoromax 3 spectrofluorometer with monochromated excitation at 390 nm. Film thickness was determined using a J. A. Woollam V-VASE spectroscopic ellipsometer by fitting the Cauchy law to data in the 900–1,600 nm non-absorbing spectral region.

Confocal microscopy was performed on a home-built setup, a schematic illustration of which is given in Supplementary Fig. 4. The sample was mounted on a piezoelectric stage that was raster-scanned with a typical integration time of 20 ms per 0.035 × 0.035 μm^2^ pixel area on the film surface. A 2.5-MHz repetition rate diode laser (PicoQuant) with ~300–500 ps pulses at 375 nm was used as excitation source. The laser light was reflected, using a long-pass dichroic mirror with reflection edge at 409 nm, onto a 100 × microscope objective (numerical aperture=0.8) that in turn focussed the light onto the sample at normal incidence, illuminating a spot with diffraction-limited diameter of 570 nm. The PL was collected through the same microscope objective and coupled into a 50-μm diameter multimode fibre that acted as the second pinhole for the confocal arrangement. The collected light was then guided onto an avalanche photodiode or into an imaging system comprising a Princeton Instruments/Acton MicroSpec 2500i spectrometer coupled to a Princeton Instruments PIXIS:100 back-illuminated camera. The PSF of the imaging system was determined experimentally to be ~340 nm; this value represents the spatial resolution that applies to PL acquisition. The PSF was deconvolved from the filtered PL intensity line profiles to obtain an estimate for the dimensions of the corresponding β-phase patterns (see Fig. 4b and Supplementary Note 1 for details).

When using the avalanche photodiode, in order to provide spectral contrast between glassy and β-phase PL emission, band-pass filters were inserted into the optical path between the fibre and the photodiode. The overlap of the PL spectra (see Fig. 1c) means that although a band-pass filter centred at 415 nm (capturing 0-0 vibronic emission from glassy PFO) rejects essentially all β-phase emission, there is not the same possibility for fully isolating the β-phase emission. The best that can be done is to place a 440-nm band-pass filter at the β-phase 0-0 vibronic peak, overlapping a trough in the glassy PFO spectrum between 0-0 and 0-1 vibronic peaks. The discrimination turns out to be rather better than it appears for these normalized spectra as there is an efficient energy transfer from glassy to β-phase chain segments; hence as the fraction of β-phase segments grows the glassy emission is rapidly quenched^7^,^8^,^12^. PL spectra in the confocal setup were acquired with the excitation laser spot centred on the required area of the film and an integration time of 15 s. All measurements were carried out at room temperature in ambient conditions.

AFM was carried out on a Park Systems NX10 microscope operating in true non-contact mode and equipped with a 300-kHz tip.

## Author contributions

D.D.C.B. initiated the project, proposed the DPN approach to conformational patterning and advised A.P. on β-phase segment formation in PFO. A.P. undertook the preliminary experiments to select suitable DPN inks, prepared the PFO films, performed the DPN writing and carried out the confocal microscopy/spectroscopy and other characterization experiments. C.R.B. assisted with optical spectroscopy measurements. S.S. and A.E.G.C. advised on DPN patterning. Y.S. and S.A.M. advised on confocal microscopy/spectroscopy and provided access to instrumentation. J.-S.K. advised on AFM measurements and provided access to instrumentation. P.N.S. provided insight on the optical characterization measurements and developed and performed the optical modelling calculations. D.D.C.B., P.N.S. and A.P. analysed the results and wrote the manuscript.

## Additional information

**How to cite this article:** Perevedentsev, A. *et al*. Dip-pen patterning of poly(9,9-dioctylfluorene) chain-conformation-based nano-photonic elements. *Nat. Commun.* 6:5977 doi: 10.1038/ncomms6977 (2015).

## Supplementary Material

Supplementary Information Supplementary Figures 1-4, Supplementary Notes 1-2, and Supplementary References
