# Supplementary material for: Dip-pen patterning of poly(9,9-dioctylfluorene) chain-conformation-based nano-photonic elements
Source: Nat Commun. 2015 Jan 19;6:5977. doi: 10.1038/ncomms6977 (PMC4309429; doi:10.1038/ncomms6977)
Supplement: Supplementary Information — Supplementary Figures 1-4, Supplementary Notes 1-2, and Supplementary References [file ncomms6977-s1.pdf]

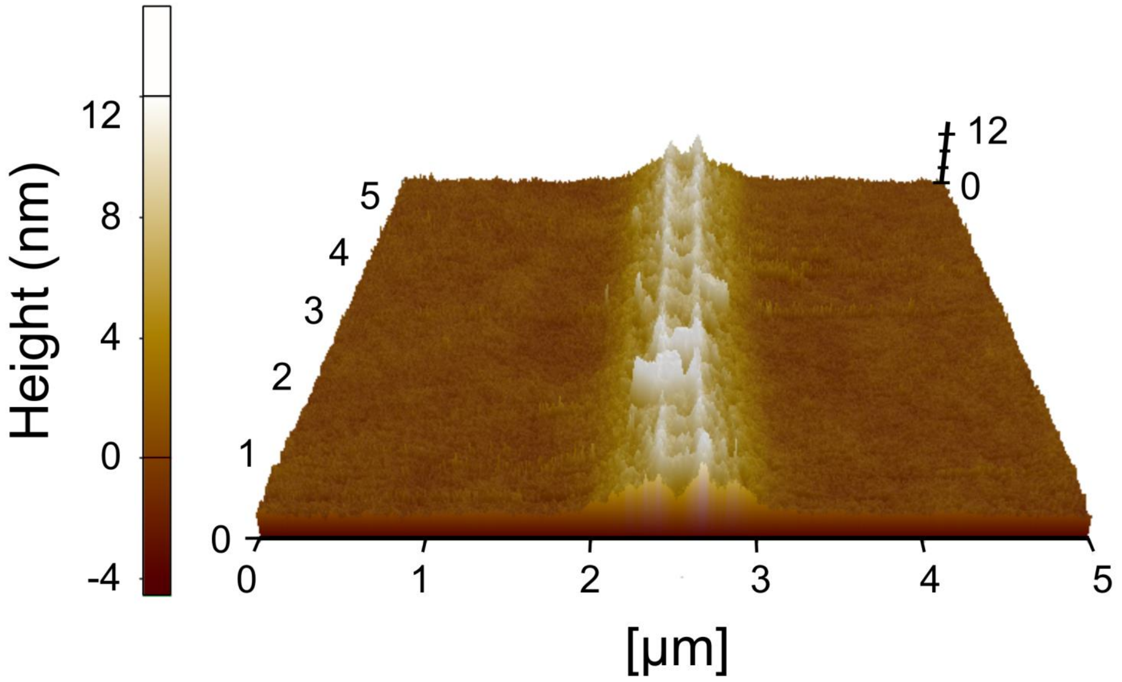

Supplementary Figure 1: DPN patterning and film surface topography. AFM image of the surface a PFO film following DPN patterning of a single continuous line at 0.1 µm/s writing speed using decalin as ink. The image corresponds to the patterned line from which the fluorescence shown in manuscript Fig. 3(a) was collected and for which the topographic profile shown in manuscript Fig. 3(b) was measured (see main text for details). The average width of the increased-height area in the AFM image corresponds closely to the deconvolved width (~1 µm) of the corresponding β-phase pattern determined by confocal PL microscopy (see manuscript Fig. 3(a)). As discussed in the main text, an increasing β-phase chain segment fraction appears to be closely linked with an increase in film roughness (see also, for example, [1]). To confirm this, we fabricated three reference PFO thin films, comprising a glassy film and two films containing approximately 6 and 32% β-phase chain segments, respectively. The corresponding RMS roughness values were 0.5, 0.6, and 9.9 nm, respectively. Where it is considered desirable, remedial action in the form of mechanical rolling or lamination of a thin over-layer could be implemented to address this issue.

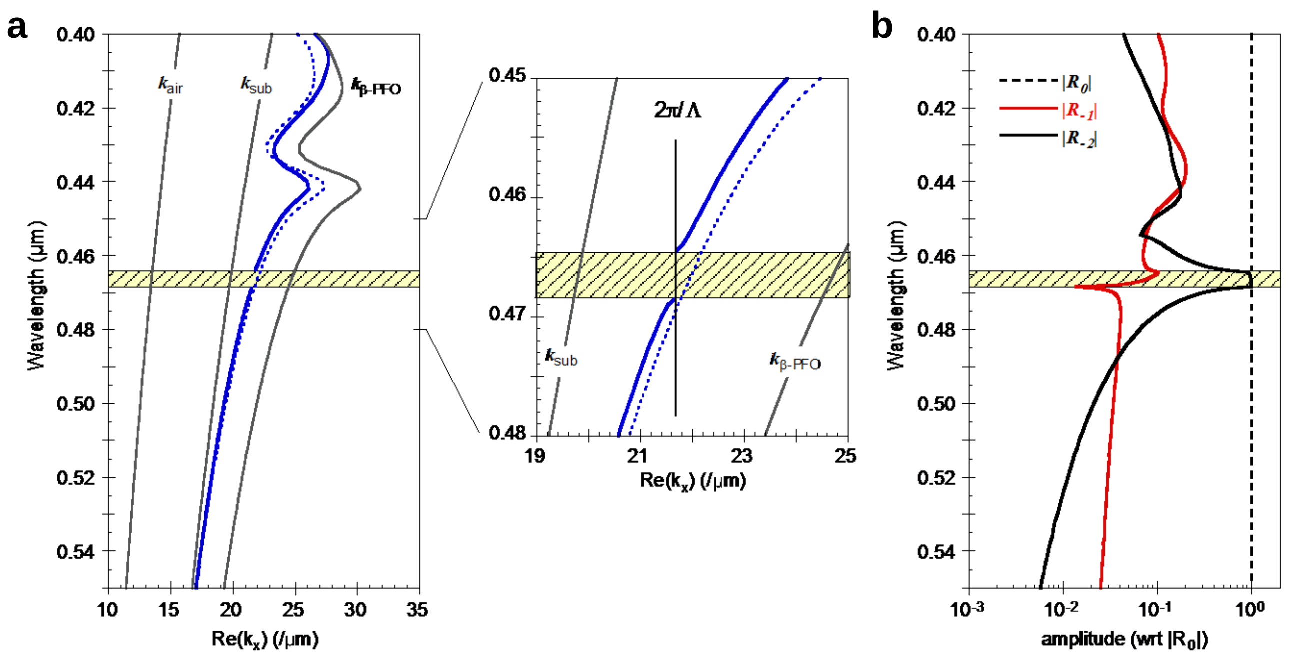

Supplementary Figure 2: One-dimensional photonic lattice calculations. (**a**) Relationship between the calculated dispersion (i.e. k_x') for the TE mode for a planar 150 nm thick film of β-phase PFO (dotted line) and the corresponding dispersion for the periodically patterned structure with alternating stripes of β- and glassy-phase PFO (solid lines); here shown in an extended scheme with the light-lines of air, the substrate and β-phase PFO. The photonic gap region (at $k_x' = (2\pi)/\Lambda$, with Λ = 290 nm) is also shown on an expanded scale. (**b**) The corresponding normalised amplitudes of the most important space harmonics. Around the photonic gap, the n = -2 harmonic momentarily acquires the same weight in the field representation as the fundamental (n = 0), illustrating the contra-coupling, while the n = -1 harmonic is barely involved.

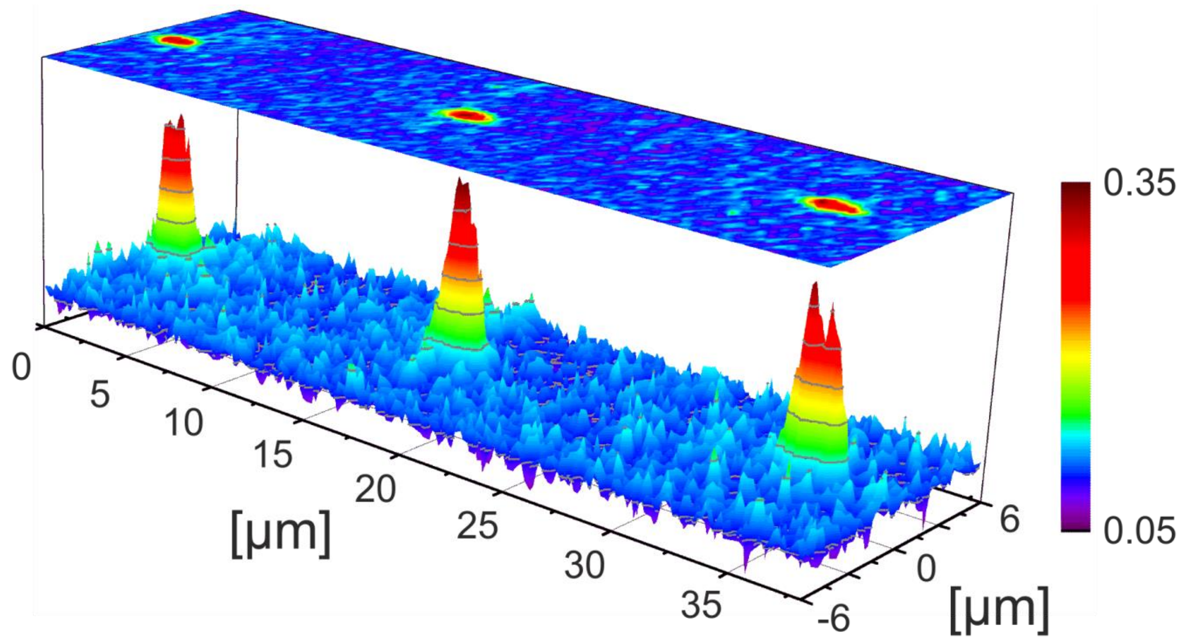

Supplementary Figure 3: DPN patterning of β-phase dot arrays. Confocal PL microscopy image of an array of β-phase dots, sequentially patterned by DPN using decalin as ink and a dwell time of 40 s. The image was recorded with PL filtered at 440 nm. The average PSF-deconvolved FWHM diameter of the β-phase dots is ~1 μm.

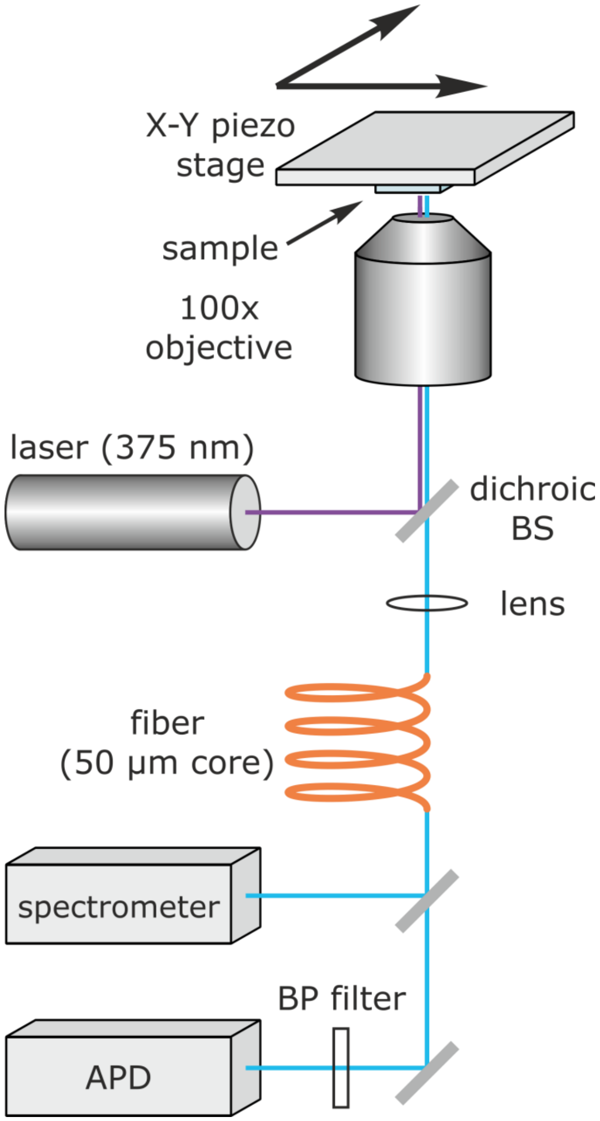

Supplementary Figure 4: Confocal PL microscopy. Schematic illustration of the confocal PL microscopy setup used to image the PL from DPN-patterned films.

Supplementary Note 1: Deconvolution of the point-spread function and estimation of the β-phase pattern dimensions.

The β-phase patterns imaged by confocal PL microscopy comprise a convolution of the "true" β-phase pattern, i.e. the area of the film containing a fraction of β-phase chain segments, with the point spread function (PSF) of the imaging system. Thus the dimensions of the "observed" β-phase pattern will invariably exceed those of the "true" pattern and, in order to estimate the "true" pattern dimensions, the PSF needs to be deconvolved from the experimental data.

The PSF of the confocal setup was determined by measuring the scattering of the excitation laser light from sub-wavelength size (~100 nm diameter) gold nanospheres. The PSF was represented by an Airy disk pattern with FWHM of its first-order maximum ≈ 340 nm. We note that these dimensions are larger than the theoretical minimum which is likely due to the relatively large size of the pinhole (fiber core diameter = 50 μm) that was required to achieve a satisfactory signal-to-noise ratio for the confocal PL microscopy measurements.

The "true" β-phase profile was assumed to be a Gaussian or a rectangle (0,1), depending on the shape of the "observed" PL intensity profile. The "true" profile was then convolved with the PSF, and the dimensions of the former were adjusted until a good fit with the experimental data was obtained. The deconvolved β-phase pattern dimensions reported in the text correspond to the FWHM of the "true" Gaussian or rectangle profiles.

As an example, PSF deconvolution is illustrated in manuscript Fig. 4(b) for the smallest imaged β-phase feature, i.e. the line written with the low boiling point ink (see main text and manuscript Fig. 4(a) for details).

Supplementary Note 2: Modelling DPN-patterned β-phase photonic element architectures.

One-dimensional, photonic lattice (grating) calculations.

The calculations behind the one-dimensional photonic crystal lattice of manuscript Fig. 5 are based on rigorous coupled wave-analysis (RCWA), one of the many well-established techniques for calculating the diffraction properties of electromagnetic waves by periodic structures (see e.g. [2–3]). Details of our specific implementation are found in [4–5]. Below we summarize the steps taken to obtain the complex propagation constants of the photonic lattice structure.

Briefly, in the half-space regions (air and substrate) a Rayleigh expansion describes the general form for the field components. All field expressions contain propagating components in the *x*-direction based on a Floquet factor and an expansion in integer multiples of the grating vector Κ, where $\mathrm{K} = (2\pi)/\Lambda$ with Λ denoting the grating period. Within the grating region, the permittivity (refractive index) varies periodically in the *x*-direction and expanded in a Fourier series, the field components are expressed with a Fourier expansion in terms of space harmonic fields. Coupled wave equations for the grating region are constructed according to Maxwell's equations using the permittivity expansion and tangential field components. Solutions for the space harmonics, representing electric and magnetic fields, are then found by solving an eigenvalue problem. Essentially at each wavelength, a full description of the electric and magnetic fields around and inside the structure may be obtained, and consequently the full scattering properties for the entire structure may be determined.

To compute the full complex photonic dispersion shown in manuscript Fig. 5 we look to find leaky-waves supported by the photonic structure with complex propagation constants, i.e. $\tilde{k}_x = k_x^{'} + ik_x^{"}$ where $k_x^{'}$ represents the propagation constant and $kx"$ is the propagation loss. More generally this may be viewed as the homogeneous problem [6], which involves computing the poles of the system, determined from the scattering matrix (S) of the structure and finding the null of the inverse S-matrix determinant**.** Numerically, however, this is notoriously an ill-posed problem, in the sense that when searching $\tilde{k}_x$– plane in the vicinity of a pole or not, this determinant is inherently close to

zero, since most of the S-matrix entries are close to zero. Following [7] we use a small S-matrix, i.e. one only accounting for propagating orders, to provide a dense matrix with a condition number between 0 and 1; consequently both the determinant and the determinant of the inverse are well behaved. Finally, since a pole of the small S-matrix is also a pole of the reflection amplitudes, we use a function based on the magnitude of the zero-order reflection amplitude $|R_0(\tilde{k}_x)|$ to solve the homogeneous problem [8]. For the root finding algorithm, in the complex $\tilde{k}_x-$ plane, a Downhill Simplex Method (DSM) is used [9].

In running the computations we found it helpful to first compute the propagating modes of a planar structure, e.g. a layer of β-phase PFO. The dispersion of the propagation constant for the planar case serves as good guess from which to start the search for $\tilde{k}_x$ for the 1-D photonic lattice in the $\tilde{k}_x-$ plane, see e.g. Supplementary Figure 2(a).

As an additional confirmation, and signature of the photonic gap, we also trace the weights of the main space harmonics, shown over the same wavelength range in Supplementary Figure 2(b) and, normalized to the zero-order magnitude. As already noted in the main text, for a 2nd order resonator we expect that all but one of the space harmonics will couple to provide the resonator action. This is indicated by $|R_{-2}| = |R_0|$ over the spectral region of the gap (the shaded region). The figure also shows the one harmonic barely involved in the contra-coupling, namely $|R_{-1}|$.

Calculations for 2-dimensional confined β-phase apertures.

Calculations in manuscript Fig. 6 are accomplished in two stages. The first is the calculation of fibre modes (here the β-phase is the fibre core, glassy phase the cladding) that follow the well-established descriptions in most undergraduate texts (see e.g. [10]). We note the full transcendental equation for the modes is used here – as opposed to linear polarized approximations. The electric field intensity for the fundamental mode (HE11), computed from all three electric field components, is shown in the inset of manuscript Figure 6(b).

The second step, which leads to the far-field radiation patterns into air [cf. manuscript Figure 6(c)], can proceed by any number of routes once the modal descriptions, or field representations of the modes, are known. One route, perhaps more suitable for a general aperture shape takes the

transverse localisation of the field intensity within a β-phase post and the Fourier de-composition of the underlying fields onto a circular polarisation mode basis into the far-field [11]. When the aperture shape is known, as in our example of a circle, a second approach and the one used for manuscript Figure 6(c), provides an analytic expression for the far-field distributions – requiring only the modal propagation constant [12]. The mode fields incident on the top surface or exit plane of the rod [cf. manuscript Figure 6(a)] are replaced by equivalent electric and magnetic current sheets; the densities of which are proportional to the tangential components of the fields of the mode. These sheets establish continuously distributed arrays of electric and magnetic dipoles that act as sources for the fields in the half space beyond the upper surface of the film.

Supplementary references

[1] Peet, J., Brocker, E., Xu, Y. & Bazan, G. C. Controlled β-phase formation in poly(9,9-di-n-octylfluorene) by processing with alkyl additives. *Adv. Mater.* **20,** 1882 (2008).

[2] Moharam, M. G., Grann, E. B., Pommet, D. A. & Gaylord, T. K. Formulation for stable and efficient implementation of the rigorous coupled-wave analysis of binary gratings. *J. Opt. Soc. Am. A* **12,** 1068 (1995).

[3] Li, L. Use of Fourier series in the analysis of discontinuous periodic structures. *J. Opt. Soc. Am. A* **13,** 1870 (1996).

[4] Stavrinou, P. N. & Solymar, L. The propagation of electromagnetic power through subwavelength slits in a metallic grating. *Opt. Comms*. **206,** 217 (2002).

[5] Stavrinou, P. N. & Solymar, L, Pulse delay and propagation through subwavelength metallic slits. *Phys. Rev. E* **68,** 066604 (2003).

[6] Nevière, M. "The homogeneous problem", chapter 5 in Petit, R. (ed.) *Electromagnetic Theory of Gratings*, Springer-Verlag, Berlin, (1980).

[7] Nevière, M., Popov, E. & Reinisch, R. Electromagnetic resonances in linear and nonlinear optics: phenomenological study of grating behavior through the poles and zeros of the scattering operator. *J. Opt. Soc. Am. A* **12,** 513 (1995).

[8] Brundrett, D. L., Glytsis, E. N., Gaylord, T. K. & Bendickson, J. M. Effects of modulation strength in guided-mode resonant subwavelength gratings at normal incidence. *J. Opt. Soc. Am. A* **17,** 1221 (2000).

[9] Press, W. H., Flannery, B. P., Teukolsky, S. A. & Vetterling, W. T. *Numerical recipes in Pascal: the art of scientific computing*, Cambridge University Press (1989).

[10] Yariv, A. *Optical Electronics*, chapter 3, 4th ed., Saunders College Publishing, Philadelphia (1991).

[11] Oulton, R. F, Stavrinou, P. N. & Parry, G. Emission from laterally confined microcavities: an optical mode approach. *Opt. Comms*. **237,** 141 (2004).

[12] Kapany, N. S. & Burke, J. J. *Optical Waveguides*, chapter 6, Academic Press, New York (1972).
